# Dataset on microplastics and associated trace metals and phthalate esters in sandy beaches of tropical Atlantic ecosystems, Nigeria

**DOI:** 10.1016/j.dib.2020.105755

**Published:** 2020-05-23

**Authors:** Omowunmi H. Fred-Ahmadu, Olusegun O. Ayejuyo, Nsikak U. Benson

**Affiliations:** a Department of Chemistry, Covenant University, Km 10 Idiroko Road, Ota, Nigeria; b Department of Chemistry, University of Lagos, Akoka, Nigeria

**Keywords:** Microplastic pollution, ATR-FTIR, Gulf of Guinea, Lagos lagoon, Beach sediment, ICP–OES

## Abstract

This article presents data on the occurrence and distribution of phthalate esters and metals associated with microplastics (MPs) (1–5 mm) collected from four beaches in the tropical Atlantic ecosystems, Nigeria, Gulf of Guinea. Information on microplastics extraction by density flotation with saturated NaCl and polymer identification with attenuated total reflectance infra-red spectroscopy (ATR-FTIR) is also provided. Analysis of six phthalate esters (PAEs) (dimethyl phthalate (DMP), diethyl phthalate (DEP), dibutyl phthalate (DnBP), benzyl butyl phthalate (BBP), di (ethyl hexyl) phthalate (DEHP), and di n-octyl phthalate (DnOP)) associated with the microplastics by performed using Gas chromatography–mass spectroscopy (GC–MS). Metals including Ag, Al, As, B, Ba, Ca, Cd, Co, Cr, Cu, Fe, K, Mg, Mn, Mo, Na, Ni, Pb, Sb, Se, Si, Sr, Ti, Tl, V, and Zn were analysed by inductively coupled plasma–optical emission spectrometry (ICP–OES). The data present the separation of microplastics from sediment, extraction with cyclohexane/ethyl acetate (1:1, v/v) and 10% HNO_3_ for phthalate esters and metals, respectively, and the determination of target analytes concentrations. The compositional distributions of MPs and levels of carcinogenic and toxic metals and phthalate esters are presented. The dataset could be used for the evaluation of ecological risk associated with PAEs in the marine ecosystems.

Specifications table

**Table:** 

Subject	Chemistry
Specific subject area	Environmental Chemistry; Analytical Chemistry
Type of data	Tables Chromatograms IR Spectra
How data were acquired	PerkinElmer Spectrum Two Attenuated Total Reflectance – Fourier Transform Infra-Red Spectrometry (ATR-FTIR) with diamond crystal, Agilent 630 Cary ATR-FTIR with diamond crystal, Agilent 7890A Gas Chromatography with Agilent 5975 Mass Spectroscopy Detector (GC–MS), Agilent 720-ES Inductively Coupled Plasma–Optical Emission Spectrometry (ICP–OES). Data was acquired and processed using Microsoft Office Excel 2016 and AddinSoft XLSTAT 2019. Analytical column was an MS C18 column (Agilent Technologies, Waldbronn, Germany) was used (450 °C: 25 m ×320 µm × 0 µm particle size).
Data format	Raw analysed
Parameters for data collection	150 beach surface sediment samples were collected. Microplastics were separated from sediment and analysed for phthalate esters (Dimethyl phthalate (DMP), Diethyl phthalate (DEP), Dibutyl phthalate (DnBP), Butyl benzyl phthalate (BBZP), di (ethyl hexyl) phthalate (DEHP), and Di n-octyl phthalate (DnOP)) and metals (Ag, Al, As, B, Ba, Ca, Cd, Co, Cr, Cu, Fe, K, Mg, Mn, Mo, Na, Ni, Pb, Sb, Se, Si, Sr, Ti, Tl, V and Zn).
Description of data collection	Microplastics were separated from sediment by density flotation with saturated sodium chloride solution and extracted with cyclohexane/ethyl acetate (1:1, v/v) by vortex mixing and sonicating for 20 min, concentration to 1 mL and analysis of extracts for phthalate esters was carried out using GC–MS. For metals analysis, microplastics were extracted with 10% nitric acid and analysed using ICP–OES.
Data source location	Lagos State/Nigeria
Data accessibility	With the article
Related research article	[1] Benson, N.U., Fred-Ahmadu, O.H. (2020). Occurrence and distribution of microplastics-sorbed phthalic acid esters (PAEs) in coastal psammitic sediments of tropical Atlantic Ocean, Gulf of Guinea, Science of the Total Environment; 10.1016/j.scitotenv.2020.139013

## Value of the data

• The dataset provides an insight into the occurrence and distribution of microplastics and associated phthalate esters (*n* = 6) and metals (*n* = 26). Both phthalate esters and some metals are inherently loaded in plastics during production however, plastics also sorb these pollutants from the environment causing elevated concentrations.
• Environmental toxicologists, governmental agencies, risk assessment bodies and environmental scientists will find the data useful for toxicological studies and dose-response determination for exposure of marine organisms to metals and phthalate esters associated with microplastics.
• This dataset is useful for further toxicological and safety investigations into the risk posed to the ecosystem and marine organisms when such microplastics are ingested.
• The data may serve as baseline concentrations of microplastics-related phthalate esters and trace metals for tropical Atlantic Ocean.

## 1 Data description

**Fig. 1:**
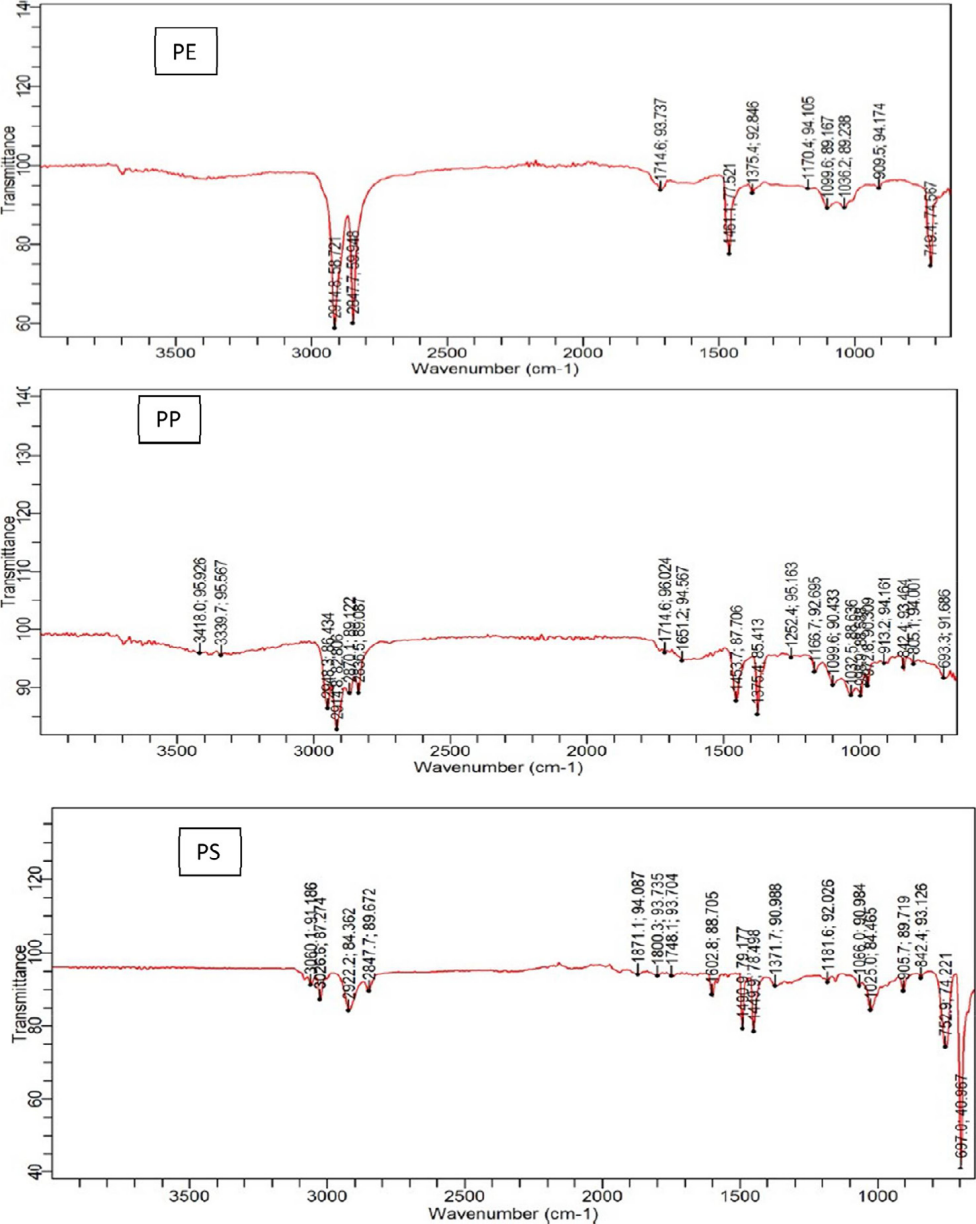
Representative spectra and absorption bands for major microplastic polymers (A) polyethylene, (B) polypropylene, and (C) polystyrene.

**Fig. 2:**
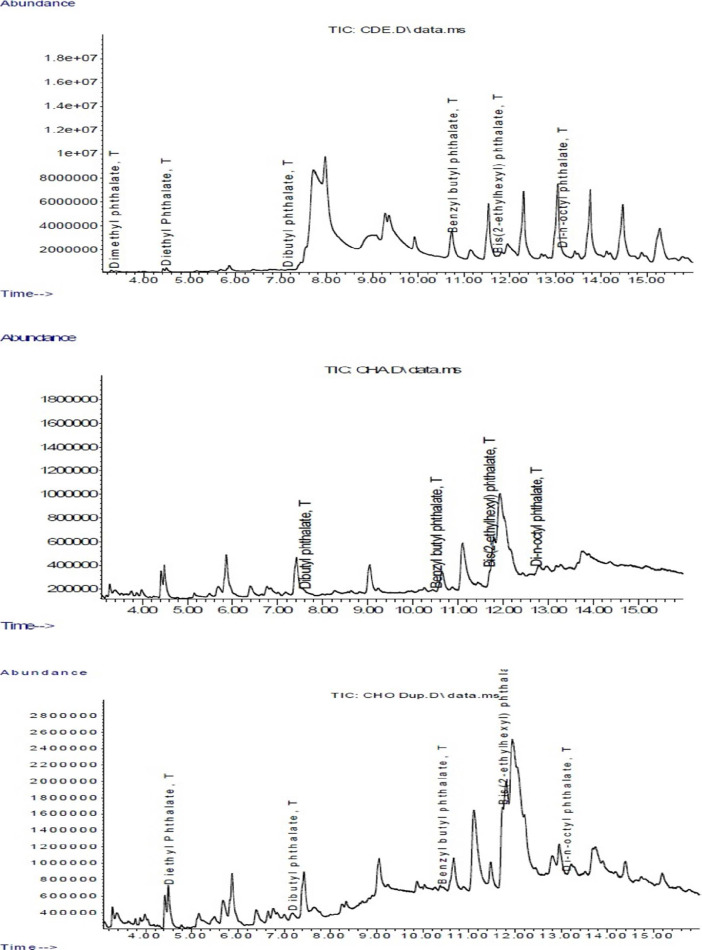
Representative chromatogram for microplastic samples phthalate esters.

Four designated sampling sites along the Nigerian coastal zone were selected for microplastic survey. The sampling codes, GPS coordinates, and site descriptions are presented in Tables 1 and 2. The quantity of microplastics according to polymeric and plastic types across the sampling locations are showed in Tables 3 and 4, respectively. However, the microplastic abundance of the different types of particles found in the sampling locations is presented in Table 5. On the other hand, Tables 6, 7, 8, and 9 presents the mean concentrations (mg/kg) of phthalate esters detected in microplastics samples collected from Oniru (O), Elegushi (E), Atican (A), and Eleko (K) beaches, respectively. There were variations across microplastics types and the different beaches sampled. The distribution and concentration of major toxic metals is shown in Tables 10, 11, 13, 14, 16, 17, 19 and 20 for Oniru, Elegushi, Atican and Eleko beaches, respectively. The distribution of other metals analysed in each of the listed beaches are shown in Tables 12, 15, 18 and 21. IR spectra showing the absorption bands representing the major polymer types found in the beaches is presented in Fig. 1. In addition, GC–MS chromatogram representing phthalate ester concentrations and distribution is depicted in Fig. 2.

**Table 1:** Coordinates of sampling locations along the coastal psammitic beaches of the study area.

	Location number	High waterline	Drift line
Oniru	1	N06°25′34.0″ E003°25′02.7″	N06°25′33.6″ E003°26′52.4″
	2	N06°25′33.4″ E003°26′53.5″	N06°25′33.2″ E003°26′53.2″
	3	N06°25′32.8″ E003°26′54.4″	N06°25′32.5″ E003°26′54.2″
	4	N06°25′31.9″ E003°26′55.9″	N06°25′31.6″ E003°26′55.7″
	5	N06°25′31.4″ E003°26′56.7″	N06°25′31.0″ E003°26′56.5″
	6	N06°25′31.2″ E003°26′.57.7″	N06°25′30.7″ E003°26′57.4″
	7	N06°25′30.9″ E003°26′58.4″	N06°25′30.3″ E003°26′58.3″
	8	N06°25′30.8″ E003°26′59.5″	N06°25′30.4″ E003°26′59.4″
	9	N06°25′30.6″ E003°26′61.1″	N06°25′30.1″ E003°26′61.2″
	10	N06°25′30.9″ E003°26′63.1″	N06°25′30.5″ E003°26′62.9″
Elegushi	1	N06°25′20.5″ E003°28′34.5″	N06°25′20.1″ E003°28.34.0″
	2	N06°25′20.8″ E003°28′33.5″	N06°25′20.5″ E003°28′33.4″
	3	N06°25′20.8″ E003°28′32.5″	N06°25′20.4″ E003°28′32.6″
	4	N06°25′20.7″ E003°28′31.8″	N06°25′20.5″ E003°28′31.8″
	5	N06°25′20.4″ E003°28′30.6″	N06°25′20.1″ E003°28′30.6″
	6	N06°25′20.0″ E003°28′29.8″	N06°25′19.7″ E003°28′29.8″
	7	N06°25′19.1″ E003°28′29.0″	N06°25′19.8″ E003°28′28.8″
	8	N06°25′20.2″ E003°28′28.1″	N06°25′20.1″ E003°28′28.1″
	9	N06°25′20.5″ E003°28′27.0″	N06°25′20.4″ E003°28′27.0″
	10	N06°25′20.8″ E003°28′25.7″	N06°25′20.9″ E003°28′25.6″
Atican	1	N06°25′28.9″ E003°35′37.9″	N06°25′28.8″ E003°35.37.9″
	2	N06°25′28.9″ E003°35′38.8″	N06°25′28.7″ E003°35′38.8″
	3	N06°25′29.0″ E003°35′.39.7″	N06°25′28.8″ E003°35′39.7″
	4	N06°25′29.0″ E003°35′40.7″	N06°25′28.8″ E003°35′40.8″
	5	N06°25′29.1″ E003°35′42.1″	N06°25′28.8″ E003°35′42.1″
	6	N06°25′29.1″ E003°35′43.5″	N06°25′28.9″ E003°35′43.6″
	7	N06°25′29.2″ E003°35′44.5″	N06°25′29.0″ E003°35′44.6″
	8	N06°25′29.3″ E003°35′45.3″	N06°25′29.1″ E003°35′45.3″
	9	N06°25′29.2″ E003°35′46.2″	N06°25′29.0″ E003°35′46.2″
	10	N06°25′29.2″ E003°35′47.3″	N06°25′29.1″ E003°35′47.3″
Eleko	1	N06°26′18.9″ E003°51′19.0″	N06°26′18.8″ E003°51.19.0″
	2	N06°26′18.9″ E003°51′18.7″	N06°26′18.7″ E003°51′18.7″
	3	N06°26′18.8″ E003°51′.18.3″	N06°26′18.8″ E003°51′18.3″
	4	N06°26′18.5″ E003°51′17.5″	N06°26′18.8″ E003°51′17.5″
	5	N06°26′18.9″ E003°51′16.9″	N06°26′18.8″ E003°51′17.9″
	6	N06°26′18.8″ E003°51′16.0″	N06°26′18.9″ E003°51′16.0″
	7	N06°26′19.0″ E003°51′15.2″	N06°26′18.0″ E003°51′15.2″
	8	N06°26’18.9″ E003°51′14.8″	N06°26′18.1″ E003°51′14.8″
	9	N06°26′18.9″ E003°51′14.3″	N06°26′18.0″ E003°51′14.3″
	10	N06°26′18.9″ E003°51′13.0″	N06°26′18.1″ E003°51′13.1″

**Table 2:** Sample codes and their descriptions for microplastic samples.

S/N	Sample code	Site description
1	AHF	Atican beach high waterline foam
2	ADF	Atican beach drift waterline foam
3	AHH	Atican beach high waterline hard plastics
4	ADH	Atican beach drift waterline hard plastics
5	OHF	Oniru beach high waterline foam
6	OHR	Oniru beach high waterline fibre/ropes
7	ODH	Oniru beach drift waterline hard plastics
8	ODF	Oniru beach drift waterline foam
9	ODR	Oniru beach drift waterline ropes/fibre
10	OHH	Oniru beach high waterline hard plastics
11	EHH	Elegushi beach high waterline hard plastics
12	EHF	Elegushi beach high waterline foam
13	EDF	Elegushi beach drift waterline foam
14	EHP	Elegushi beach high waterline pellets
15	EDH	Elegushi beach drift waterline hard
16	EDP	Elegushi beach drift waterline pellets
17	AHC	Composite high waterline sample from Atican
18	ADC	Composite drift waterline sample from Atican
19	OHC	Composite high waterline sample from Oniru
20	ODC	Composite drift waterline sample from Oniru
21	EHC	Composite high waterline sample from Elegushi
22	EDC	Composite drift waterline sample from Elegushi
23	KHC	Composite High waterline sample from Eleko
24	KHH	High waterline hard plastics from Eleko
25	KHR	High waterline fibre from Eleko
26	KDH	Drift waterline hard plastics from Eleko
27	KDR	Drift waterline fibres from Eleko
28	KDC	Composite drift waterline sample from Eleko
29	KHF	High waterline foam sample from Eleko

**Table 3:** Microplastics quantification by polymer type.

Sample sites	PE	PP	PS	PUR	EVA	PET	PA	PVC	Latex	others	Total
Oniru	879	413	322	57	16	2	33	0	3	5	1730
Elegushi	407	208	491	21	10	4	40	3	1	11	1196
Atican	38	22	123	19	9	0	8	2	0	7	228
Eleko	107	56	23	9	0	3	22	6	0	8	234

**Table 4:** Microplastics quantification by plastic types.

Sample site	Pellets	Foam fragments	Fibres	Hard fragments	Total
Elegushi	157	522	41	476	1196
Atican	5	158	14	51	228
Oniru	2	395	46	1287	1730
Eleko	3	32	19	180	234

**Table 5:** Summary of microplastics abundance in the sampling sites.

Parameters	Atican	Elegushi	Eleko	Oniru
No. of high waterline MPs	187	859	134	832
No. of drift waterline MPs	41	337	100	898
No. of current waterline MPs	0	0	0	0
No. of sediment samples	30	30	30	30
Mass (g) of MPs collected	3.45	12.62	3.11	10.78
Average no. of MPs/kg of sediment	22.8 ± 9.3	119.6 ± 38.5	23.4 ± 9.2	173.0 ± 21.3

**Table 6:** Concentration (mg/kg) of phthalate esters in Oniru beach microplastic samples (*n* = 8).

Sample code	DMP		DEP		DnBP		BBZP		DEHP		DnOP	
	Conc 1	Conc 2	Conc 1	Conc 2	Conc 1	Conc 2	Conc 1	Conc 2	Conc 1	Conc 2	Conc 1	Conc 2
ODC	0.02	0.02	0.02	0.02	1.02	1.02	0.04	0.04	19.09	19.09	0.03	0.03
OHC	BDL	BDL	0.02	0.02	4.38	4.73	0.03	0.03	45.64	44.99	0.11	0.11
ODF	0.01	0.01	BDL	BDL	1.19	1.11	0.01	0.01	8.01	8.41	0.00	0.00
ODH	0.01	0.01	0.02	0.00	3.01	3.07	0.02	0.02	8.03	8.00	0.01	0.01
ODR	0.00	0.00	0.07	0.00	1.44	1.49	BDL	BDL	6.80	6.80	0.00	0.00
OHF	0.01	0.01	BDL	BDL	BDL	BDL	0.02	0.02	3.11	3.25	0.00	0.00
OHH	0.01	0.01	0.01	0.01	BDL	BDL	0.01	0.02	11.10	11.26	0.01	0.01
OHR	0.01	0.01	0.02	0.00	BDL	BDL	0.01	0.01	4.49	4.55	0.00	0.00

BDL: Below limit of detection (LOD).

**Table 7:** Concentration (mg/kg) of phthalate esters in Elegushi beach microplastic samples (*n* = 8).

Sample Code	DMP		DEP		DnBP		BBZP		DEHP		DnOP	
	Conc 1	Conc 2	Conc 1	Conc 2	Conc 1	Conc 2	Conc 1	Conc 2	Conc 1	Conc 2	Conc 1	Conc 2
EHH	BDL	BDL	0.03	0.03	BDL	BDL	BDL	BDL	8.97	9.70	BDL	BDL
EDC	0.01	0.01	0.00	0.01	1.81	1.30	0.01	0.01	7.46	7.42	0.11	0.15
EHC	0.01	0.02	BDL	BDL	1.89	1.77	0.01	0.01	6.21	6.20	0.00	0.00
EDF	0.01	0.01	0.00	0.00	1.55	1.51	0.02	0.02	6.60	6.47	0.01	0.01
EDH	0.00	0.00	0.00	0.00	1.51	1.58	0.02	0.02	8.75	8.91	0.01	0.01
EDP	0.01	0.01	0.05	0.05	1.71	1.54	BDL	BDL	6.02	6.42	0.01	0.01
EHF	0.00	0.00	0.00	0.00	0.00	0.00	0.01	0.01	1.01	1.09	BDL	BDL
EHP	BDL	BDL	0.01	0.01	1.52	1.40	0.03	0.03	5.28	5.22	BDL	BDL

BDL: Below limit of detection (LOD).

**Table 8:** Concentration (mg/kg) of phthalate esters in Atican beach microplastic samples (*n* = 6).

Sample Code	DMP		DEP		DnBP		BBZP		DEHP		DnOP	
	Conc 1	Conc 2	Conc 1	Conc 2	Conc 1	Conc 2	Conc 1	Conc 2	Conc 1	Conc 2	Conc 1	Conc 2
ADF	15.16	15.01	0.01	0.00	BDL	BDL	0.02	0.02	6.57	6.71	0.01	0.01
ADH	0.08	0.08	BDL	BDL	1.07	0.97	0.01	0.02	7.97	7.77	0.01	0.01
AHF	BDL	BDL	0.00	0.01	BDL	BDL	5.88	6.55	4.21	4.04	BDL	BDL
AHH	0.00	0.00	0.00	0.01	1.71	1.88	BDL	BDL	7.35	7.86	BDL	BDL
ADC	0.05	0.05	0.04	0.02	BDL	BDL	0.01	0.01	4.77	4.99	0.01	0.01
AHC	BDL	BDL	BDL	BDL	0.00	0.00	0.01	0.01	5.36	5.30	0.01	0.01

BDL: Below limit of detection (LOD).

**Table 9:** Concentration (mg/kg) of phthalate esters in Eleko beach microplastic samples (*n* = 5).

Sample Code	DMP		DEP		DnBP		BBZP		DEHP		DnOP	
	Conc 1	Conc 2	Conc 1	Conc 2	Conc 1	Conc 2	Conc 1	Conc 2	Conc 1	Conc 2	Conc 1	Conc 2
KDF	0.00	0.00	0.00	0.01	0.26	0.26	BDL	BDL	2.94	2.64	0.01	0.01
KDH	BDL	BDL	BDL	BDL	0.00	0.01	0.00	0.01	0.17	0.22	BDL	BDL
KFH	BDL	BDL	BDL	BDL	0.01	0.00	0.01	0.01	0.44	0.41	BDL	BDL
KHF	0.00	0.00	BDL	BDL	1.09	1.11	0.01	0.01	4.67	4.67	BDL	BDL
KHH	0.01	BDL	BDL	BDL	1.61	1.56	BDL	BDL	4.94	4.84	BDL	BDL

BDL: Below limit of detection (LOD).

**Table 10:** Concentration (mg/kg) of major toxic metals in Oniru beach microplastic samples (*n* = 8).

Sample ID	As	Cd	Cr	Co	Cu	Fe	Pb	Ni	Zn
OHC	0.12	0.10	0.10	0.03	0.09	11.70	0.33	0.05	21.74
OHH	0.10	0.04	0.06	0.03	0.07	1.57	0.08	0.03	18.96
OHF	0.10	0.05	0.08	0.03	0.09	6.48	0.20	0.04	31.47
OHR	0.09	0.04	0.06	0.04	0.07	1.80	0.09	0.03	15.82
ODC	0.10	0.09	0.08	0.03	0.12	7.52	0.16	0.04	22.44
ODH	0.10	0.04	0.06	0.03	0.08	2.01	0.09	0.03	24.75
ODR	0.09	0.04	0.06	0.04	0.07	1.77	0.10	0.04	26.62
ODF	0.09	0.04	0.07	0.03	0.09	3.02	0.15	0.04	24.49

**Table 11:** Concentration (mg/kg) of major toxic metals in Oniru beach microplastic samples (*n* = 8).

Sample ID	Al	Mn	Sb	Ba	Mo	Se	Sr	Ti	V
OHC	5.17	5.28	0.08	0.18	0.09	0.08	1.12	0.18	0.08
OHH	1.79	0.10	0.06	0.12	0.07	0.09	0.05	0.06	0.06
OHF	4.06	0.40	0.07	0.16	0.07	0.06	0.49	0.14	0.07
OHR	1.68	0.09	0.06	0.14	0.07	0.09	0.04	0.06	0.06
ODC	3.72	0.90	0.06	2.84	0.08	0.09	0.24	0.12	0.07
ODH	2.35	0.23	0.06	0.13	0.07	0.09	0.05	0.06	0.06
ODR	2.45	0.07	0.07	0.12	0.07	0.09	0.05	0.06	0.06
ODF	2.54	0.26	0.07	0.14	0.07	0.08	0.31	0.08	0.06

**Table 12:** Concentration (mg/kg) of other metals in Oniru beach microplastic samples (*n* = 8).

Sample ID	B	Ca	Mg	K	Si	Ag	Na	Tl
OHC	0.31	7.58	7.91	3.60	6.93	0.07	45.68	0.15
OHH	0.16	6.79	2.18	1.26	0.86	0.06	19.77	0.07
OHF	0.23	7.39	21.48	5.78	3.24	0.06	49.51	0.07
OHR	0.13	6.57	2.07	1.15	1.04	0.07	16.70	0.07
ODC	0.21	7.67	6.81	3.47	3.13	0.06	37.06	0.07
ODH	0.13	7.03	3.93	1.62	0.93	0.06	20.00	0.07
ODR	0.16	7.99	4.12	1.76	1.07	0.06	14.20	0.10
ODF	0.17	7.59	19.70	2.53	2.06	0.06	25.02	0.06

**Table 13:** Concentration (mg/kg) of major toxic metals in Elegushi beach microplastic samples (*n* = 8).

Sample ID	As	Cd	Cr	Co	Cu	Fe	Pb	Ni	Zn
EHC	0.12	0.08	0.18	0.04	0.11	13.49	0.51	0.07	24.02
EHH	0.11	0.04	0.06	0.04	0.07	2.52	0.12	0.03	18.70
EHF	0.11	0.04	0.16	0.03	0.08	8.32	0.56	0.04	15.81
EHP	0.09	0.04	0.06	0.03	0.07	3.19	0.12	0.04	26.98
EDC	0.09	0.07	0.09	0.03	0.09	7.29	0.24	0.05	22.07
EDH	0.09	0.04	0.06	0.03	0.07	2.46	0.10	0.03	16.75
EDF	0.10	0.04	0.08	0.03	0.07	4.66	0.15	0.04	21.24
EDP	0.10	0.04	0.06	0.03	0.06	1.90	0.09	0.04	12.05

**Table 14:** Concentration (mg/kg) of major toxic metals in Elegushi beach microplastic samples (*n* = 8).

Sample ID	Al	Mn	Sb	Ba	Mo	Se	Sr	Ti	V
EHC	4.41	0.77	0.07	0.21	0.10	0.09	0.16	0.17	0.09
EHH	2.26	0.19	0.07	0.16	0.07	0.08	5.96	0.08	0.06
EHF	4.35	0.55	0.07	0.17	0.08	0.09	0.13	0.16	0.07
EHP	2.93	0.11	0.06	0.14	0.07	0.09	0.06	0.12	0.06
EDC	3.28	0.26	0.07	0.15	0.08	0.07	0.05	0.15	0.07
EDH	2.27	0.10	0.07	0.13	0.07	0.07	0.48	0.06	0.06
EDF	3.49	0.29	0.06	0.14	0.07	0.09	0.05	0.11	0.07
EDP	1.82	0.07	0.05	0.14	0.07	0.10	0.22	0.10	0.06

**Table 15:** Concentration (mg/kg) of other metals in Elegushi beach microplastic samples (*n* = 8).

Sample ID	B	Ca	Mg	K	Si	Ag	Na	Tl
EHC	0.43	7.80	7.64	3.41	5.28	0.07	47.57	0.09
EHH	0.21	4.81	3.36	4.34	1.37	0.07	38.27	0.10
EHF	0.27	7.73	11.65	2.84	4.75	0.07	47.42	0.08
EHP	0.14	6.48	2.71	1.69	1.56	0.06	16.18	0.09
EDC	0.20	6.10	3.91	2.50	3.27	0.06	33.15	0.05
EDH	0.15	7.56	1.58	1.26	1.07	0.06	18.71	0.08
EDF	0.15	5.01	2.99	1.85	2.18	0.07	35.36	0.06
EDP	0.13	7.61	0.92	1.04	1.18	0.07	15.25	0.07

**Table 16:** Concentration (mg/kg) of major toxic metals in Atican beach microplastic samples (*n* = 6) .

Sample ID	As	Cd	Cr	Co	Cu	Fe	Pb	Ni	Zn
AHC	0.09	0.10	0.15	0.04	0.16	4.90	0.36	0.05	19.17
AHH	0.10	0.04	0.06	0.03	0.08	1.95	0.10	0.04	25.00
AHF	0.10	0.04	0.12	0.03	0.08	4.89	0.37	0.04	25.35
ADC	0.10	0.08	0.10	0.03	0.24	4.04	0.20	0.06	14.77
ADH	0.09	0.08	0.06	0.04	0.08	1.14	0.09	0.04	19.45
ADF	0.09	0.06	0.06	0.04	0.08	1.81	0.11	0.04	24.92

**Table 17:** Concentration (mg/kg) of major toxic metals in Atican beach microplastic samples (*n* = 6) .

Sample ID	Al	Mn	Sb	Ba	Mo	Se	Sr	Ti	V
AHC	3.02	0.35	0.07	0.35	0.09	0.09	0.34	0.09	0.07
AHH	2.04	0.11	0.06	0.11	0.08	0.07	0.02	0.06	0.06
AHF	4.78	0.37	0.07	0.19	0.08	0.10	0.19	0.12	0.07
ADC	2.31	0.22	0.06	0.18	0.20	0.08	0.24	0.10	0.07
ADH	2.06	0.06	0.08	0.15	0.07	0.09	0.04	0.06	0.06
ADF	2.89	0.09	0.06	0.16	0.07	0.10	1.78	0.06	0.07

**Table 18:** Concentration (mg/kg) of other metals in Atican beach microplastic samples (*n* = 6).

Sample ID	B	Ca	Mg	K	Si	Ag	Na	T
AHC	0.58	7.67	9.19	3.72	2.16	0.07	45.72	0.10
AHH	0.16	5.75	2.15	1.72	0.94	0.06	14.98	0.11
AHF	0.19	7.60	15.25	2.79	3.29	0.06	37.74	0.10
ADC	0.48	7.91	5.66	2.40	1.85	0.07	37.96	0.08
ADH	0.14	4.82	2.11	1.32	0.97	0.06	12.49	0.10
ADF	0.15	6.97	14.73	2.91	1.26	0.06	16.67	0.09

**Table 19:** Concentration (mg/kg) of major toxic metals in Eleko beach microplastic samples (*n* = 8).

Sample ID	As	Cd	Cr	Co	Cu	Fe	Pb	Ni	Zn
KDC	0.09	0.04	0.11	0.03	0.11	4.04	2.51	0.06	19.77
KHC	0.09	0.04	0.06	0.04	0.08	1.35	0.15	0.05	21.93
KHH	0.09	0.04	0.07	0.04	0.07	1.53	0.21	0.05	17.65
KHR	0.10	0.04	0.07	0.04	0.07	1.04	0.31	0.04	18.67
KHF	0.09	0.04	0.06	0.04	0.08	1.50	0.08	0.04	18.22
KDF	0.10	0.04	0.08	0.03	0.11	4.17	0.52	0.06	23.91
KDR	0.09	0.04	0.07	0.04	0.08	1.18	1.00	0.05	18.12
KDF	0.10	0.04	0.08	0.03	0.08	3.43	0.29	0.06	20.00

**Table 20:** Concentration (mg/kg) of major toxic metals in Eleko beach microplastic samples (*n* = 8).

Sample ID	Al	Mn	Sb	Ba	Mo	Se	Sr	Ti	V
KDC	3.27	0.24	0.07	0.17	0.07	0.10	0.05	0.09	0.06
KHC	2.30	0.06	0.06	0.16	0.07	0.10	0.04	0.06	0.06
KHH	2.07	0.08	0.06	0.15	0.07	0.09	0.03	0.07	0.06
KHR	1.99	0.05	0.06	0.11	0.07	0.08	0.03	0.06	0.06
KHF	3.34	0.07	0.06	0.13	0.07	0.09	0.09	0.06	0.06
KDF	3.02	0.19	0.06	0.15	0.07	0.09	0.05	0.09	0.06
KDR	2.76	0.08	0.06	0.19	0.07	0.09	0.18	0.06	0.06
KDF	2.45	0.09	0.06	0.19	0.07	0.09	0.04	0.07	0.06

**Table 21:** Concentration (mg/kg) of other metals in Eleko beach microplastic samples (*n* = 8).

Sample ID	B	Ca	Mg	K	Si	Ag	Na	Tl
KDC	0.28	7.37	2.81	1.37	2.38	0.07	4.11	0.06
KHC	0.18	6.28	1.48	1.11	1.46	0.10	3.52	0.08
KHH	0.23	7.35	1.30	1.06	1.76	0.06	3.44	0.06
KHR	0.15	5.79	1.05	0.84	1.44	0.10	2.56	0.08
KHF	0.17	7.83	2.08	1.24	1.71	0.06	5.09	0.08
KDF	0.18	7.28	2.81	1.55	2.16	0.06	5.02	0.05
KDR	0.20	7.74	1.75	1.16	1.80	0.07	4.35	0.08
KDF	0.20	7.04	2.11	1.22	1.92	0.07	4.88	0.09

## 2 Experimental design, materials, and methods

### 2.1 Collection of samples

Sandy sediments (*n* = 150) were collected from four beaches namely Oniru, Elegushi, Atican and Eleko, in the coastal city of Lagos, Nigeria between August and November, 2019. Each beach was divided into three transects (High (HW), drift (DW), and current (CW) waterlines) and psammitic sediment samples were taken from ten points (about 100 m apart) per transect using a 0.5  ×  0.5  ×  0.2 m quadrat.

### 2.2 Sample treatment

Prior to analysis, microplastics were separated from sediment by density flotation in saturated NaCl [2], [3]–4]. Separated debris which was a mixture of plastic particles and organic matter was viewed under a stereomicroscope for selection of MPs. The selected MPs were further characterised using ATR-FTIR for polymer identification. MPs were grouped into four classes, namely hard plastics (H), foam (F), pellets (P), and fibres/ropes (R), and 0.3 g of each class was analysed accordingly. Composite samples (0.5 g), comprising a selection of all classes of MPs were also analysed. Samples were examined under a dissecting microscope, BMS 74,957 (WF10  ×  /22) at  ×  40 magnification. The categories of polymer types and physical classifications have been reported in a related study [2].

### 2.3 Sample extraction

The extraction for microplastic-sorbed phthalate esters was carried out as reported by Benson and Fred-Ahmadu [1]. Three aliquots of 5 mL of a mixture of cyclohexane (CHX) and ethyl acetate (EA), in the ratio 1:1 was added to each microplastic sample (0.25–0.5 g) in amber glass vials, previously rinsed with CHX:AE (1:1). The vials were vigorously shaken on a vortex machine and placed in an ultrasonic bath for 20 min. Then the samples were placed on an orbital rotator for 24 h to allow the samples to soak in the solvent. After centrifugation, the extract was transferred to a new vial, and the extraction process was repeated two more times. After 72 h, a total volume of 15 mL had been recovered which contained the PAE congeners adsorbed to the plastics [5]. The extract was concentrated to 1 mL, transferred to amber GC vials and stored in the fridge at 4 °C prior to GC–MS analysis. Metal extractions of microplastics was carried out two times using 10 mL of 10% HNO_3_ each time and rotating on the orbital shaker for 2 h at 150 rpm [6]. The extracts were filtered in clean glass vials and taken to ICP–OES for analysis.

### 2.4 Polymer analysis with FTIR

The resolution of the ATR-FTIR equipment was 8 cm^−1^, 32 sample scans and a range of 4000–650 cm^−1^. The absorption bands of each polymer were studied and matched with Agilent polymer ATR library with acceptable match quality set at ≥ 80% and further confirmed using validated polymer spectral data [7].

### 2.5 GC–MS and ICP–OES analysis

Sample extracts were analysed using an Agilent 7890A Gas Chromatography with Agilent 5975 Mass Spectroscopy Detector (GC–MS) for phthalate esters (in duplicates) and Agilent 720-ES Inductively Coupled Plasma–Optical Emission Spectrometry (ICP–OES) metals (in triplicates). Agilent 7890A Gas Chromatography with Agilent 5975 Mass Spectroscopy Detector (GC–MS) oven equilibration was 1 min at maximum temperature of 450 °C. The oven program was 100 °C for 0 min, 20 °C/min to 180 °C for 0 min and 10 °C/min to 280 °C for 2 min with run time set at 16 min. The carrier gas was helium and injection was by front SS splitless mode. Heat was set at 250 °C, pressure at 8.5635 psi and total flow rate at 54.659 mL/min. Septum surge flow was 3 mL/min for 2 min. The analytical column MS C18 (Agilent Technologies, Waldbronn, Germany) 25 m  ×  320 µm  ×  0 µm particle size temperature at 450 °C with initial temperature of 100 °C, pressure at 8.5636 psi, flow rate 1.6595 mL/min, average velocity of 32.774 cm/s and hold up time 1.2713 min, run time of 16 min and 1 min post run 0.57353 mL/min. The column Data acquisition and processing was carried out using Microsoft office excel and AddinSoft XLSTAT 2019. The analytes were eluted singly from the column after optimising the chromatographic parameters and their retention time obtained. Standards mix of the different phthalate esters were prepared with a concentration range of 0–100 ppm. The % recovery of matrix spikes ranged between 71.50% and 119.70%.

## Funding

This research was financially supported by Covenant University, Ota, Nigeria through CU Research Seed Grant.

## Declaration of Competing Interest

The authors declare that they have no known competing financial interests or personal relationships which have, or could be perceived to have, influenced the work reported in this article.

## Appendix Supplementary materials

Image, application 1
